# Association between salivary and blood hormone concentrations using an automated electrochemiluminescence immunoassay technique: Challenges and pitfalls

**DOI:** 10.1113/EP092542

**Published:** 2025-05-05

**Authors:** Natalie J. Hardaker, Patria A. Hume, Stacy T. Sims, Tom Stewart, Doug A. King

**Affiliations:** ^1^ Sports Performance Research Institute New Zealand (SPRINZ), Faculty of Health and Environmental Science Auckland University of Technology Auckland New Zealand; ^2^ Injury Prevention Accident Compensation Corporation Wellington New Zealand; ^3^ Traumatic Brain Injury Network (TBIN) Auckland University of Technology Auckland New Zealand; ^4^ Auckland Bioengineering Institute The University of Auckland Auckland New Zealand; ^5^ Technology and Policy Laboratory The University of Western Australia Perth Western Australia Australia; ^6^ Stanford Lifestyle Medicine Stanford University Palo Alto California USA; ^7^ School of Science and Technology University of New England Armidale New South Wales Australia

**Keywords:** electrochemiluminescence, saliva, sex hormone

## Abstract

Blood‐based measures are considered the gold standard for evaluating steroid hormone concentrations, particularly for confirmation of hormone profiles in female‐specific research. However, saliva sampling offers several advantages: it can be collected independently by participants, is less invasive and might be a more time‐ and cost‐effective solution. This preliminary study explored associations between salivary and blood plasma hormone (oestrogen, progesterone and cortisol) concentrations using an automated electrochemiluminescence immunoassay technique. Eight healthy, physically active females with a regular natural menstrual cycle provided: (1) daily app‐based menstrual tracking data; (2) daily saliva samples; and (3) two blood samples, each at different time points in the third to fifth menstrual cycle estimated using the backward calculation method. Associations between saliva and blood hormone concentrations were analysed using repeated‐measures correlations. Progesterone and oestrogen showed positive associations between blood plasma and salivary measures; (*r*_m _= 0.996, *p* < 0.0001 and *r*_m _= 0.705, *p* = 0.0507, respectively). Weak non‐significant associations for cortisol (*r*_m _= 0.245, *p* = 0.526) were found. With further work to validate the assay and develop salivary reference ranges, the electrochemiluminescence immunoassay technique might be feasible for use in quantitative determination of progesterone and oestrogen in saliva and have application in research for within‐participant monitoring of changes over time.

## Abstract (highlights)

- **What is the central question of this study?**
  Are salivary steroid hormone measures correlated with blood plasma measures using an automated electrochemiluminescence immunoassay technique?
- **What is the main finding and its importance?**
  Salivary progesterone showed a strong positive association with blood plasma measures and has potential to support monitoring of fluctuations in this hormone.

## 1 INTRODUCTION

There is a data gap in sports science and biomedical research; females have either been excluded from studies or have been included without accounting for sex in the research design or data analysis (Cowley et al., 2021). Data pertaining to the influence of hormone profiles on female athletic performance, injury risk and health outcomes are limited, often inconclusive and conflicting across different study designs (Colenso‐Semple et al., 2023). Protocols to enhance the quality of female‐specific research design, particularly related to the menstrual cycle, have been outlined (de Jonge et al., 2019). The menstrual cycle can vary within and between females (Stricker et al., 2006), making it crucial to confirm the menstrual cycle phase on the day of testing with physiological markers. For accuracy, phase verification includes a combination of the calendar‐based counting method with urinary testing for the luteinizing hormone surge and blood‐based progesterone measurement to confirm ovulation, measured as >16 nmol/L (de Jonge et al., 2019).

Given the pulsatile regulation and daily fluctuations of female sex hormones, it might be necessary to measure oestrogen (estradiol) and progesterone levels over multiple time points to monitor hormonal patterns. Daily blood sampling, or data collection, over multiple menstrual cycles have time and logistical costs for participants and a financial cost for researchers. An optimal protocol should yield good participant engagement and compliance with sample collection and provide accurate data relating to the female hormone profile. Saliva sampling could offer a non‐invasive means of hormone quantification that can be collected independently by the participant outside of the laboratory setting. The samples require minimal processing prior to laboratory analysis. The limiting factor with saliva is that the hormone concentration is much lower (only 1%–5%) than in serum, making it difficult to detect (Kozloski et al., 2014). Liquid chromatography–tandem mass spectrometry is considered the ‘gold standard’ for steroid hormone determination in blood serum when compared with immunologically based clinical methods, which are often used owing to lower costs (Handelsman, 2017).

Enzyme‐linked immunosorbent assays (ELISAs) are commonly used to measure sex hormones in blood and saliva for research and diagnostic purposes (Kozloski et al., 2014). Electrochemiluminescence immunoassay (ECLIA) has demonstrated similar or greater specificity, sensitivity and reliability when compared with other assays in the detection of steroid hormones in serum (Bolton et al., 2020; Chotboon et al., 2022) and urine (Chotboon et al., 2022). Cortisol has been validated with reference ranges of 166–507 nmol/L for serological assays and 2–22 nmol/L for salivary assays (Gagnon et al., 2018). ECLIAs for measuring estradiol and progesterone have not yet been proved in saliva.

The study aimed to determine the association between saliva and blood plasma measures of estradiol, progesterone and cortisol in healthy, physically active females using an automated ECLIA technique. The findings from this exploratory study were intended to inform whether this protocol might be used in future female‐specific research that requires the measurement of hormones over multiple time points.

## 2 MATERIALS AND METHODS

A prospective observational cohort study was undertaken. The data presented in this short communication are part of a broader exploratory study of 538 saliva samples across 31 menstrual cycles from April 2022 to March 2023, designed to test and refine the data collection protocol for future research. The small sample size of eight participants was chosen to provide insights and help ensure the robustness and effectiveness of the methodology for larger‐scale studies.

### 2.1 Ethical approval

Written informed consent was obtained from all participants. The research was conducted in accordance with the *Declaration of Helsinki*, except for registration in a database, and ethical approval was obtained from the Auckland University of Technology Ethics Committee (AUTEC #21/167).

### 2.2 Participants

Participants were recruited through the university postgraduate research school, workplaces, gyms, sports clubs and WILD AI (Wild Technologies AI Limited, London, UK) social media platforms/promotion. Eight healthy, physically active females with a regular, natural menstrual cycle (21–35 days), between 16 and 50 years of age, consented to take part in this study. Given that the study examined hormones across the menstrual cycle, several inclusion/exclusion criteria were used to ensure that other exogenous and endogenous factors did not influence the endocrine system during the 12 weeks prior to study participation. Inclusion criteria were as follows: (1) female aged ≥16 years (and had a menstrual cycle for a minimum of 2 years); and (2) had a natural regular menstrual cycle (self‐reported as 21–35 days). Exclusion criteria included: (1) unable to attend the laboratory to provide saliva and blood samples; (2) commenced any medication in the last 12 weeks that could alter reproductive hormone concentrations [glucocorticoids (e.g., prednisone), antidepressant or antipsychotic medications]; (3) thyroid disorder; and (3) current or previous brain injury.

Participants attended a 15 min introductory session with the lead researcher (N.J.H.), during which they received detailed instructions on data collection protocols and had the opportunity to ask questions.

### 2.3 Data collection methods and measures

Daily menstrual cycle data were gathered for five complete cycles, and the Low Energy in Females Questionnaire (LEAF‐Q) was administered weekly. Blood and saliva samples were collected simultaneously at two different time points during the third to fifth menstrual cycles to assess the sensitivity of the method in detecting differing hormone concentrations. Study participation concluded upon collection of the second set of samples. The two blood and saliva samples were obtained in the laboratory, whereas all other data were collected by the participant at home.

#### 2.3.1 LEAF‐Q

The LEAF‐Q was designed and validated to evaluate the physiological symptoms (e.g., fatigue, irritability) of insufficient energy intake (Melin et al., 2014). The LEAF‐Q consists of 25 items rated on a Likert scale across three categories: (1) sports injuries; (2) gastrointestinal problems; and (3) menstrual function (Supplementary Material). The LEAF‐Q was administered via the online data management platform REDCap (Research Electronic Data Capture; https://www.project‐redcap.org) (Harris et al., 2009). A unique link to the LEAF‐Q was emailed to participants every 7 days. A total LEAF‐Q score of ≥8 in combination with an injury score of ≥2 and/or menstrual dysfunction score of ≥4 was considered as having symptoms of low energy availability (LEA) (Karlsson, 2023) (the scoring key is provided in Supplementary Material). LEA is prevalent in 45% of active females (Slater et al., 2016) and is known to disrupt hormones, and these effects can occur in as little as 4 days (Areta et al., 2020). Given the length of the study, the LEAF‐Q was administered weekly to monitor LEA as a variable that could impact hormone profiles. Information from the LEAF‐Q was also used to calculate the body mass index (BMI; in kilograms per metre squared).

#### 2.3.2 Menstrual cycle tracking

‘WILD AI’ (WILD.AI Technologies Limited) is a free‐to‐download menstrual cycle tracking app with artificial intelligence (AI). Participants downloaded ‘WILD AI’, created a profile, then digitally consented to link their profile using a unique study code to the WILD AI research platform accessible by the lead researcher (N.J.H.). The data collected in WILD AI were used to estimate the timing of the expected hormone fluctuations using the backward calculation phase projection method (Gloe et al., 2023); this determined the timing of blood and saliva sample collection. Adherence to the menstrual tracking protocol was calculated by dividing the number of completion days by the total number of monitoring days (Dupuit et al., 2023). Engagement with the app was determined by cycle length difference (CLD) between consecutive cycles within each female. If the difference between the median and maximum CLD was >10, this was considered low engagement (Li et al., 2020) and might impact on result accuracy.

Cortisol follows a diurnal rhythm. In concordance with peak morning secretion (Bowles et al., 2022; Gagnon et al., 2018), on the specified days, participants attended the clinical research laboratory between 06.00 and 09.00 h in a fasted state to provide the blood and saliva samples.

#### 2.3.3 Blood and saliva sample collection

Participants provided a 2 mL saliva sample via passive drool (Supplementary Material) in a sterile cryotube (Thermo Scientific, Nunc, Biobanking and Cell Culture Cryogenic Tubes). Venous blood (∼5 mL) was collected into a BD Vacutainer Tube SST II Advance (gold) via standard venipuncture procedures. Blood samples were centrifuged (1300*g*) at room temperature (∼21°C) for 10 min. Plasma (∼1.5 mL) was aliquoted into two Eppendorf tubes. All samples were clearly labelled with the following: (1) the unique ID number; (2) the date; and (3) the exact time of the sample (e.g., F001_070223_06:25) and stored at −80°C within 30 min of collection for subsequent analysis.

### 2.4 Transfer of samples

There were two data collection sites (Auckland and Wellington, New Zealand). Samples collected and stored in Wellington were transported to the AUT Roche laboratory by air freight on dry ice 1 day prior to analysis. All blood and saliva analyses were conducted in the AUT Roche laboratory in Auckland, New Zealand.

### 2.5 Analysis of blood and saliva samples

Estradiol and progesterone were prioritized for evaluation and analysed simultaneously from the same subsample. Cortisol was measured in a second subsample that resulted in cortisol undergoing two freeze–thaw cycles. All samples were brought to room temperature overnight the day prior to analysis. Processing of saliva samples involved 500 µL of each sample being aliquoted into an Eppendorf tube and centrifuged (1500*g*) for 5 min until the supernatant was clear. The plasma and saliva samples were then transferred to Assay Cups (Hitachi, Japan) prelabelled with a unique barcode assigned to each participant. Levels of estradiol, progesterone and cortisol were measured in the plasma and saliva samples using ECLIA in the COBAS e801 analyser (Roche Diagnostics) with Estradiol III, Progesterone III (third‐generation, monoclonal antibody) and Cortisol II (second‐generation, monoclonal antibody) kits, respectively. All kits were sourced from Roche Diagnostics (NZ) and standardized before use. An external quality control was run with every batch. The duration of the automated assay was 30 min.

The quantification limits according to the manufacturer were as follows: estradiol, 18.4–11010 pmol/L; progesterone, 0.159–191 nmol/L; and cortisol, 0.054–63.4 µg/dL (Roche, 2017). These values were derived from plasma for estradiol and progesterone and from both plasma and saliva for cortisol. All values were reported in nanomoles per litre.

### 2.6 Data analysis

Data analyses were performed in R (v.4.3.0). To examine the association between blood plasma and saliva measures of estradiol, progesterone and cortisol (separately), a repeated‐measures correlation was used to account for repeated measurements per participant (i.e., two blood tests, each with a corresponding saliva sample). The repeated‐measures correlation is a statistical technique for determining the common within‐individual association for paired measures assessed on two or more occasions for multiple individuals and does not violate the assumption of independence (Bakdash & Marusich, 2017). The repeated‐measures correlations (*rmcorr*) R package was used to perform this analysis. Data were reported as correlation coefficients (*r*_m_), and a *p*‐value of <0.05 was considered statistically significant.

## 3 RESULTS

### 3.1 Participants

Of the eight participants who completed the study (Table 1), seven (87.5%) were >15 years of age at menarche. Seven participants were classified as tier one athletes, and one was classified as tier two (McKay et al., 2022). Based upon the LEAF‐Q scores, no study participants met the criteria for being ‘at risk’ of LEA, and this did not change over the study. There was good average adherence and engagement from all participants who completed the study. Sixteen saliva samples and 16 plasma matched samples were analysed (i.e., two blood and two saliva samples per participant).

**TABLE 1:** Participants age, height, weight, calculated body mass index, reported exercise, menstrual cycle length and LEAF‐Q score by mean with SD and range of reported characteristics.

Characteristic (*n* = 8 participants)	Mean ± SD	Range
Age, years	39.4 ± 5.8	32–47
Height, cm	170.0 ± 3.7	165–176
Weight, kg	64.8 ± 6.6	58–79
BMI, kg/m^2^	22.4 ± 2.5	20–27
Exercise, h/week	6.6 ± 3.4	2.5–11
Menstrual cycle length, days	26.9 ± 4.4	17–42
Adherence	79.8 ± 26.1	39–100
Engagement^a^	1.6 ± 1.7	0–5
LEAF‐Q score	3.4 ± 2.4	0–9

*Note*: Adherence was calculated as the number of days completing the symptom report divided by the number of days in the study (Dupuit et al., 2023).

Abbreviations: BMI, body mass index; CLD, cycle length difference; LEAF‐Q, Low Energy in Females Questionnaire.

^a^ Engagement was calculated as maximum CLD − median CLD (if >10 = poor engagement) (Li et al., 2020).

### 3.2 Correlation of blood plasma and salivary measures

**FIGURE 1:**
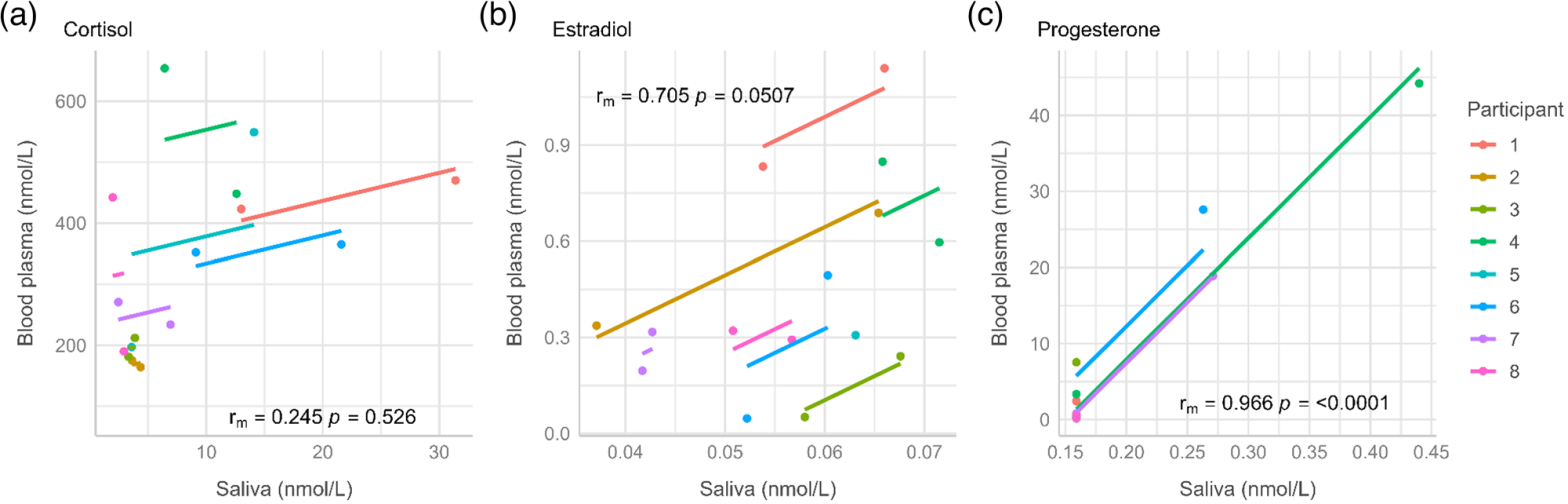
Repeated‐measures correlation plots showing the association between saliva and blood measures of cortisol (a), estradiol (b) and progesterone (c), in *n* = 8 female athletes.

The plasma hormone concentrations for the eight participants were within normal ranges. In comparing the 16 saliva samples with the 16 matched blood plasma samples, there was a non‐significant weak correlation for cortisol (*r_m_* = 0.245, *p* = 0.526; Figure 1a). There were strong positive correlations between blood plasma and salivary measures of progesterone (*r*_m _= 0.966, *p* ≤ 0.0001; Figure 1c) and estradiol (*r*_m _= 0.705, *p* = 0.0507; Figure 1b). The *rmcorr* coefficient (*r*_m_) represents the strength of the shared intra‐individual association between blood plasma and salivary measures. The parallel lines represent the line of best fit (or not) between individual data points for each participant and the common regression slope.

## 4 DISCUSSION

The findings of this study were encouraging. Salivary progesterone showed a strong positive correlation with blood plasma measures. This was consistent with other work (Kozloski et al., 2014) highlighting that, although salivary immunoassays for evaluation of progesterone need further validation, there is value in refining these methods. The weaker correlation observed between measures of serum and salivary estradiol in this study was consistent with a previous study (Kozloski et al., 2014). The concentration of estradiol in the saliva is only ∼2%–5% of that measured in serum, and the lowest estradiol concentrations are lower than the lowest progesterone concentrations across the menstrual cycle (Stricker et al., 2006); therefore, the lower analyte concentrations are harder to detect and require a higher specificity of the assay to minimize interference from other substances in the sample. There are well‐documented challenges with using immunoassays to measure hormone concentrations even in serum. This is particularly evident in low‐estradiol states (e.g., follicular phase, puberty, in males) (Lynch et al., 2014). Studies investigating the predictive value of salivary or serum estradiol have highlighted a low correlation and a likely upward bias of estradiol concentration in the follicular (low‐oestrogen) phase of the menstrual cycle (Warade, 2017).

It was anticipated that there would be a stronger correlation between plasma and salivary cortisol measures, given that the assay has been validated for use in both fluids and given that salivary cortisol is widely used for research and diagnostic purposes. In a meta‐analysis of previous studies in adults using immunoassays, higher correlations between serum and saliva have been reported (*r *= 0.43 to *r *= 0.82) when evaluated using Pearson's correlation or Spearman's ρ (Koksal, 2021). The lower correlation value (*r*_m _= 0.245, *p* = 0.526) in the present study is likely to be attributed to limited power rather than a flaw in the repeated‐measures statistical approach, which is an important component of menstrual cycle‐related research (Schmalenberger et al., 2021). Exploratory Pearson correlations on averaged trials (*r* = 0.61, *p* = 0.1055) and first trials (*r* = 0.387, *p* = 0.343) of data in this study suggest a moderate relationship, with non‐significance, further supporting this point. Previous studies used different techniques, including radioimmunoassay and ELISA (Koksal, 2021), which might contribute to the different correlation values recorded. There is also limited research in females (Konishi et al., 2014), suggesting that there are inter‐individual differences and there might be high‐ and low‐‘secretors’ of salivary progesterone and cortisol. The concentration of these hormones in saliva differs after correcting for blood hormone values.

### 4.1 Benefits of the ECLIA compared with other assay techniques

Although other methods of evaluating hormone levels exist, many of them are serum based (Handelsman, 2017; Kozloski et al., 2014). There are practical advantages of ECLIA; these include the wider linear range, which allows for single‐dilution measurements of concentration, a smaller sample volume requirement for analysis, and the automated multiplex testing platforms that support higher throughput, which is more efficient when processing large numbers of samples (Bolton et al., 2020). ECLIA has also shown higher sensitivity and specificity than ELISA, making it potentially more suitable for detecting low concentrations of analyte (Bolton et al., 2020; Chotboon et al., 2022). All these features are particularly beneficial for ‘in‐field’ research methodologies; the ability to collect multiple samples over time allows for real‐time monitoring of hormone fluctuations, and participants can self‐collect samples, without the need to visit a laboratory.

### 4.2 Challenges and pitfalls

Menstrual cycle phase was estimated using the backward calculation phase projection method (Gloe et al., 2023). The use of this method limits the accuracy of phase determination and does not allow confirmation of ovulation. Although participants in the present study were provided with careful instructions about sample collection and storage, there was no direct oversight of this from the research team. Poor sample collection can impact on the quality of the sample. A further challenge to sample quality is salivary flow rate (calculated by dividing the volume of the sample by the time taken to obtain it; in millilitres per minute), which has been inversely associated with total protein concentration (Walsh et al., 2004). Both these factors can affect the measurement of some target analyte concentrations (Beltzer et al., 2010; Hofman, 2001). Owing to the self‐collect protocol used in this study and the fact that previous work has indicated that flow rate and total protein concentration does not affect steroid hormone measurement in saliva, flow rate was not measured. Cortisol was analysed from a subsample that had undergone a second freeze–thaw cycle; as a result, the quality of both samples might have been compromised or, given that salivary cortisol has been shown to be stable for up to four freeze–thaw cycles (Sontag et al., 2023) and that plasma might be more vulnerable to freeze–thaw cycles (Mitchell et al., 2005), it could be that saliva and serum responded differently to the second freeze–thaw cycle. To minimize the risk of this occurring, it is recommended that, where possible, at the point of collection the samples are aliquoted prior to freezing. Although this was intended as an exploratory study, the small sample size must also be considered when interpreting these findings.

## 5 CONCLUSION

Analysis of saliva samples using an automated ECLIA technique detected changes in progesterone concentration across the menstrual cycle and showed a strong association with serum measures. In a research context, salivary progesterone has the potential to support monitoring of changes and fluctuations in sex hormones. This could improve applied and in‐field methodologies when blood sampling is not feasible or when high‐frequency sampling is required and increases participant burden. There is value in further work with larger studies of female participants to refine and validate this technique for female‐specific research, in order that reliable, relevant measures are accessible to participants and can be adhered to easily.

## AUTHOR CONTRIBUTIONS

The work was carried out in the Auckland University of Technology, Roche laboratory. Natalie Hardaker: Conceptualization; methodology; resources; investigation; formal analysis and data curation; writing—original draft; writing—review and editing. Patria Hume: Conceptualization and methodology; formal analysis and data curation; writing—review and editing. Stacy Sims: Conceptualization and methodology; formal analysis and data curation; writing—review and editing. Tom Stewart: Formal analysis and data curation; writing—review and editing. Doug King: Writing—review and editing. All authors approved the final version of the manuscript and agree to be accountable for all aspects of the work in ensuring that questions related to the accuracy or integrity of any part of the work are appropriately investigated and resolved. All persons designated as authors qualify for authorship, and all those who qualify for authorship are listed.

## CONFLICT OF INTEREST STATEMENT

The authors declare that there are no competing interests associated with the research contained within this manuscript. The opinions expressed are those solely of the authors and do not necessarily reflect those of the Accident Compensation Corporation, New Zealand.

## Supporting information



Supplementary material

Supplementary material

## Data Availability

The data that support the findings of this study are available from the corresponding author upon reasonable request.
