# Supplementary material for: Association between salivary and blood hormone concentrations using an automated electrochemiluminescence immunoassay technique: Challenges and pitfalls
Source: Exp Physiol. 2025 May 5;110(12):1795–801. doi: 10.1113/EP092542 (PMC12665927; doi:10.1113/EP092542)

**Supplementary material**

LEAF_Q questions and instructions.

The low energy availability in females questionnaire (LEAF –Q), focuses on physiological symptoms of insufficient energy intake. The following pages contain questions regarding injuries, gastrointestinal and reproductive function. We appreciate you taking the time to fill out the LEAF-Q and the reply will be treated as confidential.

Unique Study Code:

Address:

E-mail:

Mobile:

Occupation:

Education:

Age: (years)

Height: (cm)

Weight: (kg)

Your highest weight with your present height: (kg) (excluding pregnancy)?

Your lowest weight with your present height: (kg)?

What is your desired weight (kg)?

Do you smoke?

- Yes
- No

Do you use any medication (excluding oral contraceptives)?

- Yes
- No

If yes, what kind of medication?

Your normal amount of training (average) – number of hours per week and what kind of exercise, such as running, swimming, cycling, strength training, technique training etc. (‘training’ is any kind of physical activity)

Comments or further information regarding exercise:

1. Injuries

A: Have you had absences from your training, or participation in competitions during the last year due to injuries?

- No, not at all
- Yes, once or twice
- Yes, three or four times
- Yes, five times or more

A1: If yes, for how many days absence from training or participation in competition due to injuries have you had in the last year?

- 1-7 days
- 8-14 days
- 15-21 days
- 22 days or more

A2: If yes, what kind of injuries have you had in the last year?

Comments or further information regarding injuries:

2. Gastrointestinal function

- A: Do you feel gaseous or bloated when you do not have your period?
- Yes, several times a day
- Yes, several times a week
- Yes, once or twice a week or more seldom
- Rarely or never

B: Do you get cramps or stomach aches that are not related to your menstruation?

- Yes, several times a day
- Yes, several times a week
- Yes, once or twice a week or more seldom
- Rarely
- Never

C: On average how often do you have bowel movements?

- Several times a day
- Once a day
- Every second day
- Twice a week
- Once a week or more rarely

D: How would you describe your normal stool?

- Normal (soft)
- Diarrhoea-like (watery)
- Hard and dry

Comments regarding gastrointestinal function:

3.1 Use of contraceptives

A: Do you use oral contraceptives?

- Yes
- No

A1: If yes, why do you use oral contraceptives?

- Contraception
- Reduction of menstruation pains
- Reduction of bleeding
- To regulate the menstrual cycle in relation to performances etc.
- Otherwise, menstruation stops
- Other

A2: If no, have you used oral contraceptives in the past?

- Yes
- No

A2:1: If yes, when and for how long?

B: Do you use any other kind of hormonal contraceptives? (e.g., hormonal implant or coil)

- Yes
- No

B1: If yes, what kind?

- Hormonal patches
- Hormonal ring
- Hormonal coil
- Hormonal implant
- Other

3.2 Menstrual function

A: How old were when you had your first period?

- 11 years or younger
- 12-14 years
- 15 years or older
- I don’t remember
- I have never menstruated (If you have answered “I have never menstruated” there are no further questions to answer)

B: Did your first menstruation come naturally (by itself)?

- Yes
- No
- I don’t remember

B1: If no, what kind of treatment was used to start your menstrual cycle?

- Hormonal treatment
- Weight gain
- Reduced amount of exercise
- Other

C: Do you have normal menstruation)

- Yes
- No (go to question C6)
- I don’t know (go to question C6)

C1: If yes, when was your last period?

- 0-4 weeks ago
- 1-2 months ago
- 3-4 months ago
- 5 months ago or more

C2: If yes, are your periods regular? (Every 28th to 34th day)

- Yes, most of the time
- No, mostly not

C3: If yes, for how many days do you normally bleed?

- 1-2 days
- 3-4 days
- 5-6 days
- 7-8 days
- 9 days or more

C4: If yes, have you ever had problems with heavy menstrual bleeding?

- Yes
- No

C5: If yes, how many periods have you had during the last year?

12 or more

- 9-11
- 6-8
- 3-5
- 0-2

C6: If no or “I don’t remember”, when did you have your last period?

- 2-3 months ago
- 4-5 months ago
- 6 months ago or more
- I am pregnant therefore I do not menstruate

D: Have your periods ever stopped for 3 consecutive months or longer (besides pregnancy)?

- No, never
- Yes, it has happened before
- Yes, that’s the situation now

E: Do you experience that your menstruation changes when you increase your exercise intensity, frequency or duration?

- Yes
- No

E1: If yes, how? (Check one or more options)

- I bleed less
- I bleed fewer days
- My menstruations stops
- I bleed more
- I bleed more days

**Supplementary material**


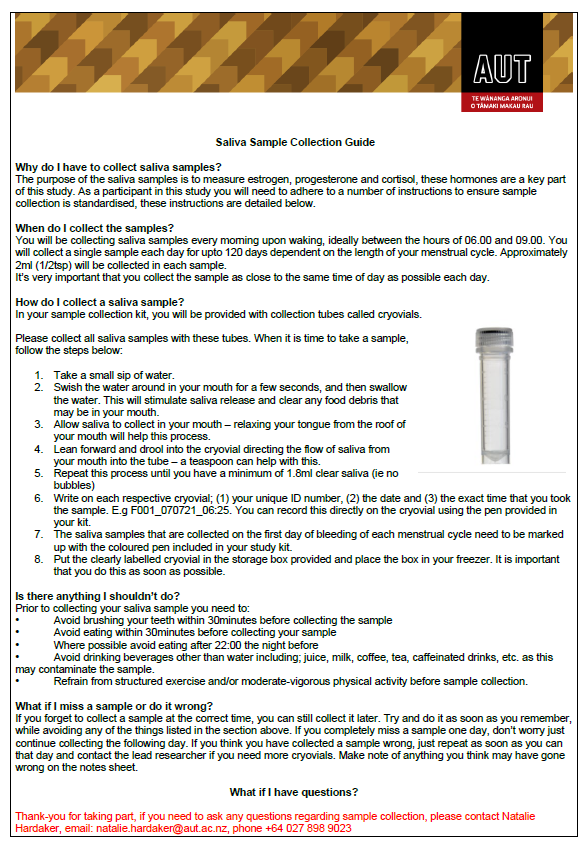

Supplement: Supplementary file 1 — Supplementary material [file EPH-110-1795-s002.docx]
