# Supplementary figures and images for: Association between salivary and blood hormone concentrations using an automated electrochemiluminescence immunoassay technique: Challenges and pitfalls
Source: Exp Physiol. 2025 May 5;110(12):1795–801. doi: 10.1113/EP092542 (PMC12665927; doi:10.1113/EP092542)

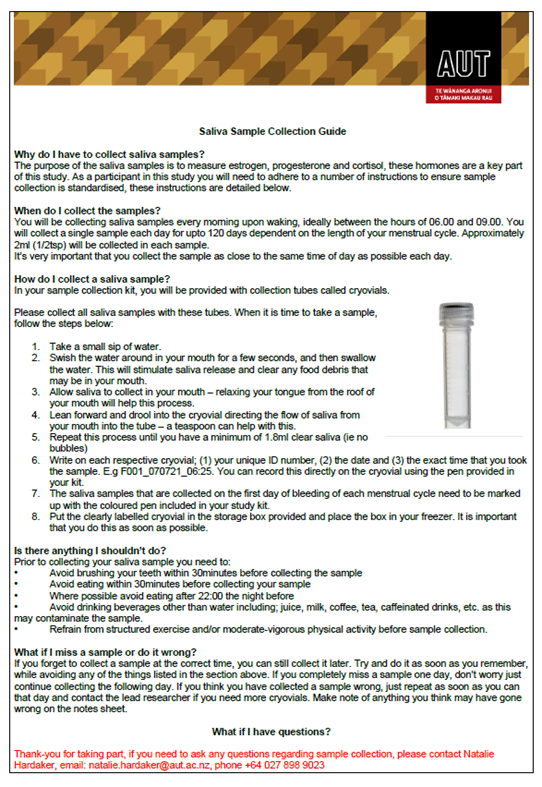

Supplement: Supplementary file 2 — Supplementary material [file EPH-110-1795-s001.png]
